# HIGHER VALUES IN LIVER ELASTOGRAPHY AND MELD SCORE ARE MORTALITY PREDICTORS ON LIVER TRANSPLANT WAITING LIST

**DOI:** 10.1590/0102-672020180001e1360

**Published:** 2018-06-21

**Authors:** Lucas Souto NACIF, Denise C PARANAGUA-VEZOZZO, Alina MATSUDA, Venancio Avancini Ferreira ALVES, Flair J CARRILHO, Alberto Queiroz FARIAS, Luiz Carneiro D’ALBUQUERQUE, Wellington ANDRAUS

**Affiliations:** 1 Liver and Gastrointestinal Transplant Division, Department of Gastroenterology, School of Medicine, University of São Paulo; 2 Department of Pathology, School of Medicine, University of São Paulo, São Paulo, SP, Brazil.

**Keywords:** Liver transplantation, End-stage liver disease, Logistic models, Survival analysis., Transplante de fígado, Estágio final da doença hepática, Modelo para estágio final da doença de fígado, Regressão logística, Sobrevida de pacientes.

## Abstract

**Background:**

Liver elastography have been reported in hepatocellular carcinoma (HCC) with higher values; however, it is unclear to identify morbimortality risk on liver transplantation waiting list.

**Aim:**

To assess liver stiffness, ultrasound and clinical findings in cirrhotic patients with and without HCC on screening for liver transplant and compare the morbimortality risk with elastography and MELD score.

**Method:**

Patients with cirrhosis and HCC on screening for liver transplant were enrolled with clinical, radiological and laboratory assessments, and transient elastography.

**Results:**

103 patients were included (without HCC n=58 (66%); HCC n=45 (44%). The mean MELD score was 14.7±6.4, the portal hypertension present on 83.9% and the mean transient elastography value was 32.73±22.5 kPa. The median acoustic radiation force impulse value of liver parenchyma was 1.98 (0.65-3.2) m/s and 2.16 (0.59-2.8) m/s in HCC group. The HCC group was significantly associated with HCV infection (OR 26.84; p<0.0001), higher levels of serum alpha-fetoprotein (OR 5.51; p=0.015), clinical portal hypertension (OR 0.25; p=0.032) and similar MELD score (p=0.693). The area under the receiver operating characteristics (AUROC) showed sensitivity and specificity for serum alpha-fetoprotein (cutoff 9.1 ng/ml), transient elastography value (cutoff value 9 kPa), and acoustic radiation force impulse value (cutoff value 2.56 m/s) of 50% and 86%, 92% and 17% and 21% and 92%, respectively. The survival group had a mean transient elastography value of 31.65±22.2 kPa vs. 50.87±20.9 kPa (p=0.098) and higher MELD scores (p=0.035).

**Conclusion:**

Elastography, ultrasound and clinical findings are important non-invasive tools for cirrhosis and HCC on screening for liver transplant. Higher values in liver elastography and MELD scores predict mortality.

## RESUMO

**Racional:**

A elastografia hepática tem sido relatada nos carcinomas hepatocelulares (CHC); porém, não é claro identificar o risco de morbimortalidade na lista de transplante hepático.

**Objetivo:**

Avaliar a morbimortalidade com elastografia transitória e escore MELD.

**Método:**

Pacientes adultos com cirrose na triagem para transplante de fígado foram incluídos no estudo.

**Resultados:**

Foram incluídos 103 pacientes (sem CHC n=58 (66%), CHC n=45 (44%). O escore MELD médio foi de 14,7±6,4, a hipertensão portal foi de 83,9% e o valor médio de elastografia transitória foi de 32,73±22,5 kPa. O valor médio de ARFI (Impulsão de Força de Radiação Acústica) do parênquima hepático foi de 1,98 (0,65-3,2) m/s e 2,16 (0,59-2,8) m/s no grupo CHC. O grupo CHC foi significativamente associado à infecção por vírus da hepatite C (OR 26,84, p<0,0001), níveis mais altos de alfa-feto proteína sérica (OR 5,51; p=0,015), hipertensão portal clínica (OR 0,25; p=0,032) e pontuação MELD semelhante (p=0,693). Os valores de AUROCs (Area Under the Receiver Operating Characteristics) mostraram sensibilidade e especificidade para a alfa-feto proteína sérica (limite de 9,1 ng/ml), valor elastografia transitória (valor de corte 9 kPa) e valor ARFI (valor de corte 2,56 m/s) de 50% e 86%, 92% e 17% e 21% e 92%, respectivamente. O grupo de sobrevivência apresentou valor elastografia transitória médio de 31,65±22,2 kPa vs. 50,87±20,9 kPa (p=0,098) e valores mais elevados de MELD (p=0,035).

**Conclusão:**

Valores mais elevados na elastografia do fígado e nos escores MELD predizem a mortalidade.

## INTRODUCTION

Hepatocellular carcinoma (HCC) is the most important malignant liver tumor with increasing incidence and prevalence in the Western countries, mainly related to chronic hepatitis C infection and subsequent development of liver cirrhosis^16, 18)^. As a result of surveillance programs aimed at identifying HCC at early stages, many patients have been eligible for curative interventions such as liver transplantation (LT) ^18)^. The current guidelines of the European Association for the Study of the Liver and the American Association for the Study of Liver Diseases recommend LT as the preferred alternative in most patients within the Milan criteria ^2^ ^,^ ^6^ ^,^ ^11^. 

Over the last decade, it was noted the development and improvement of methods for predicting the degree of fibrosis and cirrhosis using non-invasive methods, and there are important scores and biomarkers of fibrosis that help the identification of the clinical cirrhosis stages^1^. These methods are based on image as unidimensional ultrasound with transient elastography (FibroScan)^5^, as well as ultrasound bidimensional in elastometry by ARFI (Acoustic Radiation Force Impulse)^3^ ^,^ ^19^. But, on another way, the real and potential role of these methods on advanced and complicated liver chronic disease is not yet clear. Recent studies have reported ultrasound-based techniques such a shear wave-based elastography like dispersion ultrasonic vibrometry or elastography point quantification capable useful predictor of malignancy and to screen for hepatocellular carcinoma. However, the data on the stiffness of HCCs and their background livers are controversy^9^. 

In this scenario, the progression of fibrosis and the onset of cirrhosis are important prognostic factors for patients on the liver transplantation waiting list. The majority of complications and mortalities are related to the severity of cirrhosis, and portal hypertension and its associated complications ^14^ ^,^ ^**21**^. The validation of the ability of elasticity quantification to reveal in real life, liver stiffness as an important factor for its widespread application. 

The objective of this study was to show the ability to perform an early diagnosis and choose the best treatment to improve the long-term survival rates and identify morbimortality risk on liver transplantation waiting list. Furthermore, this study aimed to assess liver stiffness, ultrasound and clinical findings in cirrhotic patients with and without HCC on screening for liver transplant.

## METHODS

This study was approved by the Institutional Review Board with number 279.864 on May 22 of 2013 fulfilling all requirements for prospective studies in humans, following the guidelines of the 1975 Declaration of Helsinki.

From October 2012 to December 2013, a total of 103 consecutive adult patients (68% men, mean age 52±11.5 years) due to end-stage liver diseases were submitted to clinical, radiological, elastography and laboratorial assessment. 

The following variables were analyzed: 1) demographics parameters: age, gender, etiology of the liver disease (viral/non-viral); 2) clinical features: significant portal hypertension (defined as a hepatic vein pressure gradient (HPVG) ≥10 mmHg or the presence of gastro-esophageal varices, splenomegaly (>12 cm) with platelets count <100,000 cells/mm3, and need for diuretics to control ascites}. When these criteria were present but hepatic portal vein measurement was not available portal hypertension clinically-based was diagnosed), Child-Turcotte-Pugh (A/B/C); 3) radiological findings and Doppler ultrasound 4) serum laboratory analysis: bilirubin (mean and median values), alpha fetoprotein (were compared according the cut off value obtained by the area under the receiver operating characteristics (AUROC) curve and values ≥20 or <20 values); 5) elastography findings (mean, median values and the cut off value) comparison with Model for End-stage liver disease (MELD) score; and 6) morbidity and mortality (comparison with MELD score).

### Elastography (FibroScan and ARFI)

An important goal of this study was to perform and to compare data from transient elastography (TE) acquired by FibroScan^TM^ (Echosens, France) and Acoustic Radiation Force Impulse (ARFI) as Virtual Touch^TM^ (Siemens Acuson S2000, Germany) in the groups with and without HCC according the recent EASL/AASLD diagnostic guideline for HCC. Was used Acoustic Radiation Force Impulse (ARFI) to compare the liver stiffness between tumoral and non-tumoral liver parenchyma.

#### *Elastography technique*

The TE was performed with the patient placed in a bed after 2 h of fasting in the supine position with the right arm upright and under the head. The transducer gently touched the patient’s skin, in the right intercostal space between the 9^th^ and 10 ribs, preferably choosing the reference position located at the intersection between the xyphoid and the median axillary lines at the point where hepatic biopsy is usually recommended. 

The screen with three ultrasound signal evaluations had to show a regular liver wave in M mode, a linear A signal away from the large vessels and an elastogram equivalent to a finger´s width. The images were obtained when the operator observed the best conditions, as mentioned earlier. After 10 shots or 60% successful shots and an interquartile range (IQR) less than 30%, was considered valid measurement. The results were registered in numbers and expressed in kilopascals (kPa). The possible values in TE range from 2-75 kPa

Following TE, ARFI was performed at the same intercostal point, and the exam was performed by the same experienced operator (with over 1000 exams performed). Were collected 10 measurements in the same place that the region of interest (ROI) was found, and the interquartile range was observed to obtain 30% less than the median final value, according to the European Association for the Study of the Liver Diseases (EASL) and World Federation for Ultrasound in Medicine and Biology (WFUMB) guidelines^3^ ^,^ ^5^. Results of ARFI are reported in meters per second at a range of between 0.5 to 4.0 m/s.

There were relatively few unreliable ARFI results, although considered, because the interquartile range variation is usually larger in complicated cirrhosis, according to a recently published paper^7^ ^,^ ^22^. This finding could be related to the presence of HCC alongside heterogeneous cirrhosis.

### Inclusion and exclusion criteria

Adult patients with cirrhosis undergoing screening for HCC or on the LT waiting list were considered consecutively for inclusion. Informed consent was obtained from all participants. Patients with problematic anatomy, such as those with narrow intercostal spaces and small right hepatic lobes (n=1), high body mass indices (n=2), ascites (n=4), nodule diagnosed as something other than HCC (n=5) or a success rate <60% in 10 measurements on Fibroscan examination (n=16), were all excluded. Were excluded 28 patients out of 131 and included 103 patients in this study. Were excluded all patients with possible acute alcoholic hepatitis.

#### *Statistical analysis*

Median values (25% quantile - 75% quantile) were presented for quantitative variables and percentages for qualitative variables. Mann-Whitney test was performed to evaluate the difference between median from two groups and Fisher test was considered to verify the association. Furthermore, ANOVA or Kruskal-Wallis followed by non-parametric Turkey test were applied to find differences among groups and was performed simple and multiple logistic regression with statistical program in R, version 2.15.1. Finally, ROC Curve allowed to finding cutoffs for exams. A value of p<0.05 was considered significant in the final analysis. Follow-up was performed from the date of the first exam session until death, the last visit or the date of LT. Follow-up was continued until December 31, 2013. Clinical examination and laboratory data collection were routinely performed. 

## RESULTS

### Clinical and demographic parameters

Baseline characteristics of the enrolled patients are presented in Table 1. The median age was 55 years old (20-77), and 70/103 (68%) patients were male. The etiology was hepatitis C, ethanol, hepatitis B, NASH and miscellaneous etiologies in 35%, 29%, 8%, 7% and 21% of patients, respectively. The presence HCC in patients with hepatitis C had significant statistical finding (p<0.0001). Thirty-eight (38%) patients were CPT A, 47 (47%) were CPT B and 15 (15%) were CPT C. The mean MELD score was 14,7 (±6) and median was 14 (6-32). The BMI score mean was 28 (±6). The ethnicities were white, brown, black and yellow; with respectively 69%, 24%, 6% and 1%. The encephalopathy was presented in 27%. The significant portal hypertension (PH-CB) was presented in 84% of the patients and these finding had significant difference in groups with and without HCC (p=0.038).

**TABLE 1:** Baseline characteristics of the enrolled patients

Parameters	Total 103 (100%)	HCC 45 (44%)	Non-HCC 58 (66%)	p
Age (y)	53±11.5	54.1±12.2	52.1±11.0	0.448
Gender (M/F) %	68/32	69/31	68/32	>0.05
Etiology % HCV OH HBV NASH Cryptogenic CBS Auto immune Others	35 29 8 7 6 5 3 7	51 4 18 4 4 2 7 5	21 50 0 9 7 7 0 7	<0.001
MELD score Mean Median	14.7±6.4 14 (6-32)	14.7±7.0 12 (6-30)	14.7±6.0 14 (6-32)	0.693
CPT A/B/C %	38/47/15	43/45/12	35/50/15	0.801
AFP (≥20 ng/ml) %	25.7	38.9	10.3	0.011
AFP (≥9.1 ng/ml) %	33.3	50	13.7	0.003
PH-CB %	83.9	73.6	91.7	0.038

Mean and standard deviation; numbers and percentage; PH-CB=portal hypertension clinically-based; MELD= Model for End Stage Liver Disease; AFP=alpha fetoprotein; HCC=hepatocellular carcinoma; CPT =Child-Turcotte-Pugh; HCB=hepatites C vírus infection; NASH=nonalcoholic steatohepatitis; OH=alcoholic hepatitis; HBV=B virus infection; CBS=secondary biliary cirrhosis

### Serum laboratory analysis

The median serum alpha fetoprotein (AFP) level was 3.75 ng/ml (0.6-32150). Serum alpha fetoprotein levels were higher than 9.1 ng/ml (33.3%) in 34 patients and higher than 20 ng/ml (25.7%) in 26. The median serum alpha fetoprotein in HCC group was 7.2 ng/ml (0.6- 32150) higher than no HCC group 2.9 ng/ml (0.9-226), (p=0.02).

The median platelets level was 98 x 10^3^/mm^3 )^(13-339) and INR median was 1.25 (0.89-3.29). The median total bilirubin level was 2.03 mg/dl (0.27- 15.76) and the median albumin level was 3.5 g/l (1.8-4.9).

### Radiological characteristics (ultrasound and Doppler)

A single nodule was observed in 27 patients (25%) and 2, 3 or more nodules in 18 (17%). The average size of the primary tumor was 3.5 cm in diameter (0.6-13 cm) at diagnosis.

The median spleen diameter was 32 cm (10-108). Splenomegaly (spleen higher than 20 cm) was presented in 86 patients (85%). Portal vein thrombosis was presented in 11 (36 % partial portal vein thrombosis and 64 % complete thrombosis). The average speed of portal vein was higher than 13 cm/s in 69.6% patients. Recanalization of paraumbilical vein was presented in 29 % and ascitis in 49% with significant difference in both groups (p=0.045).

### Elastography findings

The elastography findings (ARFI and FibroScan) were shown in Table 2. The median FibroScan value was 26.3 kPa (3.8-75), in the HCC group this value was 24,6 kPa (4.6-75) and non-HCC group was 33.3 kPa (3.8-75) (p>0.05, Table 2).

**TABLE 2:** Elastography findings of the enrolled patients

Parameters	Total 103 (100%)	HCC 45 (44%)	Non-HCC 58 (66%)	p
FibroScan (kPa) Mean Median	32.73± 22.5 26.3 (3.8-75)	30.4± 21.0 24.6 (4.6-75)	35.6± 23.9 33.3 (3.8-75)	0.491
ARFI (m/s) Mean Median	2.02± 0.59 2.17(0.65-3.4)	1.97±0.64 1.98(0.65-3.2)	2.06±0.54 2.21(0.88-3.4)	0.565
ARFI lesion(m/s) Mean Median	NA NA	1.89±0.74 2.16(0.59-2.8)	NA NA	NA
ARFI soma(m/s) Mean Median	2.79± 1.15 2.51(0.88-5.5)	3.57±1.13 3.7(1.27-5.51)	2.06±0.54 2.21(0.88-3.4)	<0.001

Mean and standard deviation; numbers and percentage; MELD=Model for End Stage Liver Disease; HCC=hepatocellular carcinoma; NA=not applicable

The FibroScan values of the HCV and ethanol etiologies were statistically different among groups without HCC (p=0.022), whereas the liver stiffness of the ethanol etiology was higher, approximately double the value of elastography in HCV etiology (Table 3).

**TABLE 3:** Comparison between Fibroscan® and ARFI® liver stiffness of ethanol (OH) and HCV etiology among groups with and without HCC and MELD score analysis

Parameters/Groups	HCC HCV (n=21)	Non-HCC HCV (n=9)	p
Fibroscan^®)^ (kPa) MELD	30.89±4.09 **14.28±6.60**	22.1±4.04 **11.63±4.41**	0.353 0.312
ARFI^®)^ (m/s)	2.14±0.12	2.06±0.09	0.979
Parameters/Groups	OH (n=1) HCC	OH (n=18) non-HCC	p
Fibroscan^®)^ (kPa) MELD	26.6±NA **21**	44.88±5.62 **12.18±(5.0)**	0.647 NA
ARFI^®)^ (m/s)	2.31±0.43	2.21±0.11	0,81
Parameters/Groups	Non-HCC HCV (n=9)	Non-HCC OH (n=18)	p
Fibroscan^®)^ (kPa) MELD	22.1±4.04 **11.63**±4.41	44.88±5.62 **12.18±(5.0)**	**0.022** 0.48
ARFI^®)^ (m/s)	2.06±0.09	2.21± 0.11	0.961

Mean and standard deviation; MELD=Model for End Stage Liver Disease; HCC=hepatocellular carcinoma; NA=not applicable

The most prevalent hepatopathy with higher mortality was due alcohol in 55% (n=5), followed by HCV (n=2), cryptogenic in one case, and also chronic liver disease in only one case. The average value found in this population by FibroScan was 50.87 kPa and by the ARFI was 2.02 m/s. The patients who earlier died in this follow up had high values ​​in elastography and in MELD score (p<0.05, Table 4)

**TABLE 4:** Transient elastography (Fibroscan), ARFI and MELD score assessing mortality

Parameters	Death	live (n=63)	p
Fibroscan^®)^ (KPa) n=4 ARFI^®)^ (m/s) n=9 MELD score n=9	**50.87±20.9** 2.02±0.37 21.00±6.09	31.65±22.2 2.02±0.57 14.01±6.25	**0,098** 0,994 **0,035**

Mean and standard deviation; MELD=Model for End Stage Liver Disease; HCC=hepatocellular carcinoma; ARF= acoustic radiation force impulse; KPa=Kilo Pascal; NA=not applicable

### Accuracy diagnosis

The accuracy diagnosis showed an improvement when the TE value was combined with ARFI values and serum AFP levels. The ROC curves showed the diagnostic accuracy with best cut-off value for FibroScan (9 kPa), ARFI (2.56 m/s) and AFP (9.1 ng/ml), with a sensitivity of 92% on Fibroscan and a specificity of 92% on ARFI. These accuracy diagnosis parameters demonstrate an increased risk of HCC when a TE cut-off was higher than 9 kPa in general and 26.3 kPa in HCV patients. A cut-off for ARFI of 2.56 m/s included a higher percentage of HCC patients, around 73%. 

### Logistic Regression

Regression analysis for HCC detection was performed in the univariate analysis and the following variables were significant different between HCC and non-HCC group: gender, ethnicity, symptoms, ascitis, encefalopaty, Portal hypertension clinically-based, diagnosis and elastography (Table 5). On the multiple regression analysis, only three variables statistically correlated with the presence of HCC: serum alpha fetoprotein level >20 ng/ml, diagnosis, and hepatitis C virus etiology with the highest odds ratio (OR,Table 5). 

**TABLE 5:** Regression analysis for HCC detection: simple and multiple logistic regression analysis

	Variables	Odds Ratio	95% CI	p
Simple analysis	HCV etiology	26.84	5.44-132.35	<0.0001
	Others etiology	16.48	3.41-79.51	<0.0001
	Ascitis	0.42	0.18-0.93	0.033
	AFP (≥20 ng/ml)	5.51	1.4-21.69	0.015
	Encephalopathy	0.76	0.31-1.85	0.546
	PH-CB	0.25	0.07-0.89	0.032
	Fibroscan (>9 kPa)	1.75	0.45-6.8	0.419
	ARFI (>2.56 m/s)	2.78	0.66-11.69	0.162
Multiple analysis		Odds Ratio	95% CI	p
	HCV etiology	12.06	2.03-71.78	0.006
	AFP (≥20 ng/ml)	5.47	1.16-25.75	0.032

HCV=hepatitis C virus; PH-CB=portal hypertension clinically-based; AFP=alpha-fetoprotein; HCC=hepatocellular carcinoma

### Endpoints

The study concluded after 12 months, or if the patient dropped out (34 patients dropped out, 14 with unfavorable clinical conditions, 10 with low MELD score and two were with tumor drop out Milan criteria and one loss of clinical follow up); died (n=9) or underwent definitive clinical intervention (LT in seven with 86% of one year survival rate) prior to this. The patients were then grouped according to mortality; 63/103 patients (65%) survived in follow up on the 12 months, while 9/103 (8.7%) did not. The most prevalent hepatopathy for mortality was alcoholic cirrhosis at 55% (n=5), followed by HCV (n=2), cryptogenesis in one case, and chronic liver disease, also in only one case. A further 31 dropped out or underwent LT. The survival group had a mean Fibroscan score of 31.65±22.2 kPa, while those who died showed a mean score of 50.87±20.9 kPa. The difference between the means is very close to statistical significance (p=0.098, Table 4).

## DISCUSSION

The present study confirms that increased liver stiffness, serum alpha fetoprotein level, hepatitis C virus etiology and portal hypertension clinically-based (PH-CB) are predictors of the presence of HCC in liver cirrhosis patients. This study is a pioneer in the evaluation of patients by liver elastography in screening program for liver transplantation with severe liver disease patients, with higher clinical portal hypertension and almost all cases with class Child-Turcotte-Pugh B/C liver function. So, the importance of this study is to highlight that increased liver stiffness is associated with higher mortality but not well in real life to predict HCC.

Liver stiffness measurement using transient elastography is recognized as accurately able to assess the stage of liver fibrosis in patients with chronic hepatitis C virus infection^8^ ^,^ ^22^, even as was observed in this study. But the main importance of this cohort is that almost 84% of the patients had significant portal hypertension and advanced chronic liver disease. Moreover, the transient liver elastography shows high values in both HCC and cirrhosis livers and does not help to differentiate between them.

Patients with higher values of elastography measurements that mainly reflect the liver fibrosis progression of the cirrhotic patients are associated with HCC^7^. Therefore, it is also supposed that elastography measurements might be a valuable noninvasive tool for assessing the presence and risk of developing HCC. To our knowledge, first studies reported a cut-off value of 53.7 kPa as suggestive for the presence of HCC in HCV cirrhotic patients^7^. However, in this cohort, was found a lower cut-off value by Fibroscan of 26.3 kPa in HCV patients and 9kPa in the general group with increased risk of HCC.

Masuzaki et al.^10^ observed that patients with chronic HCV liver disease stratified by elastography were presented variable risk of HCC per year depending on the value obtained by the TE method^10^. In this study, was also observed that higher liver stiffness get by TE and ARFI methods associated with increased values ​​of serum AFP and etiology of hepatitis C virus (HCV) were positive predictors to the presence of hepatocellular carcinoma (HCC) in patients with liver cirrhosis. Therefore, risk factors for the development of hepatocellular carcinoma in these patients were the presence of hepatitis C virus, the serum AFP with cutoff point higher than 9.1 ng/dl and ​​elastography values greater than 9kPa in FibroScan and higher than 2.56 m/s in ARFI.

Trinchet et al.^20^ conducted a prospective, randomized controlled trial with ultrasound every 3-6 months and they found a higher number of false positives in the lower range arm, and also recommended reduced the intervals in suspected smaller lesion every three months^20^. In our clinical service, we followed a total of 884 cirrhotic patients by ultrasound and AFP serum were analyzed at least one time per year during 5-10 years and they observed the presence of HCC in 72 cases (8.1%) and an annual incidence rate of HCC of 2.8%^15^. Thus, probably patients with alcoholic cirrhosis should remain in a surveillance every six months, but in patients with hepatitis C, when they get by TE value higher than ​​26.3 kPa, it is indicated a tracking every three months due to the higher risk of developing HCC.

The advanced fibrosis and cirrhosis are different among the various etiologies^**17**^, and this statement has been confirmed by the literature over the years, clearly demonstrated by pioneering study in late 1950’s with assessments based on analysis of 10,000 consecutive autopsies^4^ ^,^ ^12^. In our study, was compared the elastography values ​​of patients without HCC in ethanol and HCV etiology, and was observed the double values ​​obtained by FibroScan (p=0.022) with similar CHILD and MELD. This suggests that for the same degree of liver function among HCV and ethanol etiologies, the ethanol hepatopathy have more liver stiffness, most because of more fibrosis and therefore it showed higher values ​​in elastography.

The value of MELD score is well established as prognostic evaluation in patients with cirrhosis and in list for liver transplantation. In Brazil, this score was implemented in 2006 for organ allocation, and was demonstrated retrospectively by us an increase in the number of liver transplants performed in School of Medicine, University of São Paulo from 2002 to 2012^13^. We noted in this study that the value of MELD score was significantly higher in patients with cirrhosis assessed by elastography who died (p=0.035) and higher values ​​for liver elastography (FibroScan) with a significant trend (p=0.098) in these same patients, demonstrating a positive relationship with mortality.

There were several limitations of this study. For example, we had a selection bias due to all our patients were in cirrhosis or severe end of liver disease. Also, to get a good correlation of non-invasive elastography and clinical data to detect HCC is necessary to validate with a higher number of cases. However, we presented two independent risk factors for hepatocellular carcinoma like the HCV etiology and increased serum levels of AFP were demonstrated. We could also identify a positive correlation between cases with increased values ​​of MELD score and the values ​​of FibroScan in patients who died. 

The potential clinical benefit of this study with liver elastography measurements, Doppler ultrasound and clinical findings in these patients is that patients with HCV and HCC should be prioritized when liver stiffness is 26>kPa and higher values could predict morbimortality. This could alter organ allocation priorities and improve clinical management with more prospective imaging on waiting lists. A longer, better-designed prospective study is needed before concrete recommendations for change can be proposed.

## CONCLUSIONS

The elastography is an important non-invasive tool for monitoring severe cirrhosis and may help in management of hepatocellular carcinoma with the association of serum alpha fetoprotein, clinical, laboratory and imaging findings. However, liver elastography shows higher values in both HCC and cirrhosis livers. Finally, the evaluation of elastography showed that liver elastography and MELD scores can predict risk of mortality.
